# Understanding Housing Stability for Child Welfare-Involved Families

**DOI:** 10.23889/ijpds.v9i5.2887

**Published:** 2024-09-18

**Authors:** Sydney Johnson, Kristine Piescher, Leah Lindstrom Rhea, Amy Stetzel

**Affiliations:** 1 Center for Advanced Studies in Child Welfare, University of Minnesota; 2 Corporation for Supportive Housing

## Objective and Approach

Prior research has found that experiences of homelessness are associated with an increased likelihood of child protection involvement, and vice-versa. However, it is not clear how involvement with the child welfare system may impact family housing stability during and after their involvement. The objective of this study was to more deeply understand the housing stability of families involved with child welfare over time.

To examine this issue, we linked administrative data from Minnesota’s Homelessness Management Information System with social services data from the Department of Human Services (e.g., receipt of food support, childcare or housing assistance, child welfare), for families involved with child welfare in Minnesota between 2019 and 2021.

## Results

Approximately a quarter of child welfare-involved families experienced homelessness during their involvement with the child welfare system. Rates of homelessness were similarly high in the two years prior to and following child welfare involvement. Although a large proportion of families experienced homelessness before and during their child welfare involvement, we saw no increase in the proportion of families receiving housing support after their involvement in the system.

## Conclusions and Implications

Our findings indicate that involvement with the child welfare system and the resources provided through this involvement do not have a significant impact on family’s experiences of homelessness. This suggests the need for closer collaboration between the child welfare system and housing services to identify more effective solutions for housing instability among families within the child welfare system.

